# DUSP7 inhibits cervical cancer progression by inactivating the RAS pathway

**DOI:** 10.1111/jcmm.16865

**Published:** 2021-08-26

**Authors:** Huimin Bai, Meiying Song, Ruili Jiao, Weihua Li, Jing Zhao, Meizhu Xiao, Mulan Jin, Zhengyu Zhang, Haiteng Deng

**Affiliations:** ^1^ Department of Obstetrics and Gynecology Beijing Chao‐yang Hospital Capital Medical University Beijing China; ^2^ Department of Obstetrics and Gynecology Fuxing Hospital Capital Medical University Beijing China; ^3^ Department of Obstetrics and Gynecology Beijing Chaoyang District Maternal and Child Health Care Hospital Beijing China; ^4^ Department of Pathology Beijing Chao‐yang Hospital Capital Medical University Beijing China; ^5^ MOE Key Laboratory of Bioinformatics School of Life Sciences Tsinghua University Beijing China

**Keywords:** biological characteristics, cervical cancer, DUSP7, lentiviral vector, proteomics, RAS pathway

## Abstract

To determine the differentially expressed proteins (DEPs) between paired samples of cervical cancer (CC) and paracancerous tissue by quantitative proteomics and to examine the effects of DUSP7 expression on the tumorigenesis and progression of CC. Proteomic profiles of three paired samples of CC and paracancerous tissue were quantitatively analysed to identify DEPs. The relationship between DEP expression and patient clinicopathological characteristics and prognosis was evaluated. The effects of the selected DEPs on CC progression were examined in SIHA cells. A total of 129 DEPs were found. Western blot and immunohistochemistry (IHC) staining analyses confirmed the results from quantitative proteomic analysis showing that the selected DEP, HRAS, P‐ERK1/2, and PLD1 levels were increased, whereas the DUSP7 level was decreased in CC tissue compared with the paired normal paracancerous tissues. The IHC results from the CC TMA analysis showed that the decreased expression of DUSP7 (*p* = 0.045 and 0.044) was significantly associated with a tumour size >2 cm and parametrial infiltration. In addition, the decreased expression of DUSP7 and increased expression of p‐ERK1/2 were adversely related to patient relapse (*p* = 0.003 and 0.001) and survival (*p* = 0.034 and 0.006). The expression of HRAS and p‐ERK1/2 was decreased in DUSP7‐SIHA cells compared with NC‐SIHA cells (*p* = 0.0003 and 0.0026). Biological functions in vitro, including invasion, migration and proliferation and tumour formation in vivo were decreased in DUSP7‐SIHA cells (all *p* < 0.05) but increased in shDUSP7‐SIHA cells (all *p* < 0.05). DUSP7 inhibits cervical cancer progression by inactivating the RAS pathway.

## 1 INTRODUCTION

Human cervical carcinoma (CC) is the third most common cancer worldwide and is the most frequent gynaecological cancer in developing countries. China has approximately 150,000 new cases of CC each year, and CC tends to occur in younger people ^1^. CC remains a serious health problem in women. It is therefore crucial to explore the pathogenesis of CC and discover effective therapeutic targets for this lethal disease. The key aetiological role of human papillomavirus (HPV) in the development of CC and its precursors has been well documented ^2^, and the use of HPV vaccination in women has the potential to reduce the incidence of CC in the future. However, due to the high price of the vaccine, coverage rates are still low, especially in low‐ or middle‐income countries ^3^. Furthermore, viral presence is not sufficient to induce CC ^4^, suggesting that a distinct molecular mechanism could play a key role in its transformation and progression.

Proteomics is defined as the comprehensive global analysis of a specific proteome, the set of all proteins expressed in a cell or a biological system or organism at a given point in time and under certain conditions. Proteomics has been widely used to identify certain proteins with complex biological functions related to the pathogenesis of various diseases, including human malignancies. Wang et al. ^5^ compared the proteomes of the primary tumours of CC patients with and without lymph node metastasis and revealed that patients with high FABP5, HspB1 and MnSOD expression have a high risk of lymph node metastasis and adverse prognosis. By comparing the proteomes of primary CC tissues and corresponding adjacent normal tissues, Zhang et al ^6^ found that Notch signalling, viral carcinogenesis, RNA transport and Jak‐STAT signalling play an important role in tumour progression. In these studies, the study and control groups had the same genetic backgrounds. Differentially expressed proteins (DEPs) identified through this manner may reflect the process of tumorigenesis and progression of CC to some extent. However, no further details on the preparation of tissue specimens were provided. In this analysis, the tissue specimens available for proteomic examination were subjected to a stricter pathological evaluation so the results would more accurately reflect the factors involved in the invasion and progression of cervical cancer. The protein profiles between paired samples of CC and paracancerous tissues were compared and analysed, with the goal of providing useful information about diagnostic biomarker or molecular therapeutic targets for patients with CC.

## 2 MATERIALS AND METHODS

### 2.1 Materials and cell culture

The main materials, including reagents, instruments and antibodies for Western blot (WB) and immunochemistry (IHC), used in this work are shown in Table S1 and S2, respectively. The human CC cell line SIHA was cultured in RPMI‐1640 supplemented with 10% foetal bovine serum (FBS) and antibiotics (100 U/ml penicillin and 100 μg/ml streptomycin) at 37°C in a humidified atmosphere in a 5% CO_2_ incubator.

### 2.2 Specimen collection of CC and paracancerous tissue

**FIGURE 1:**
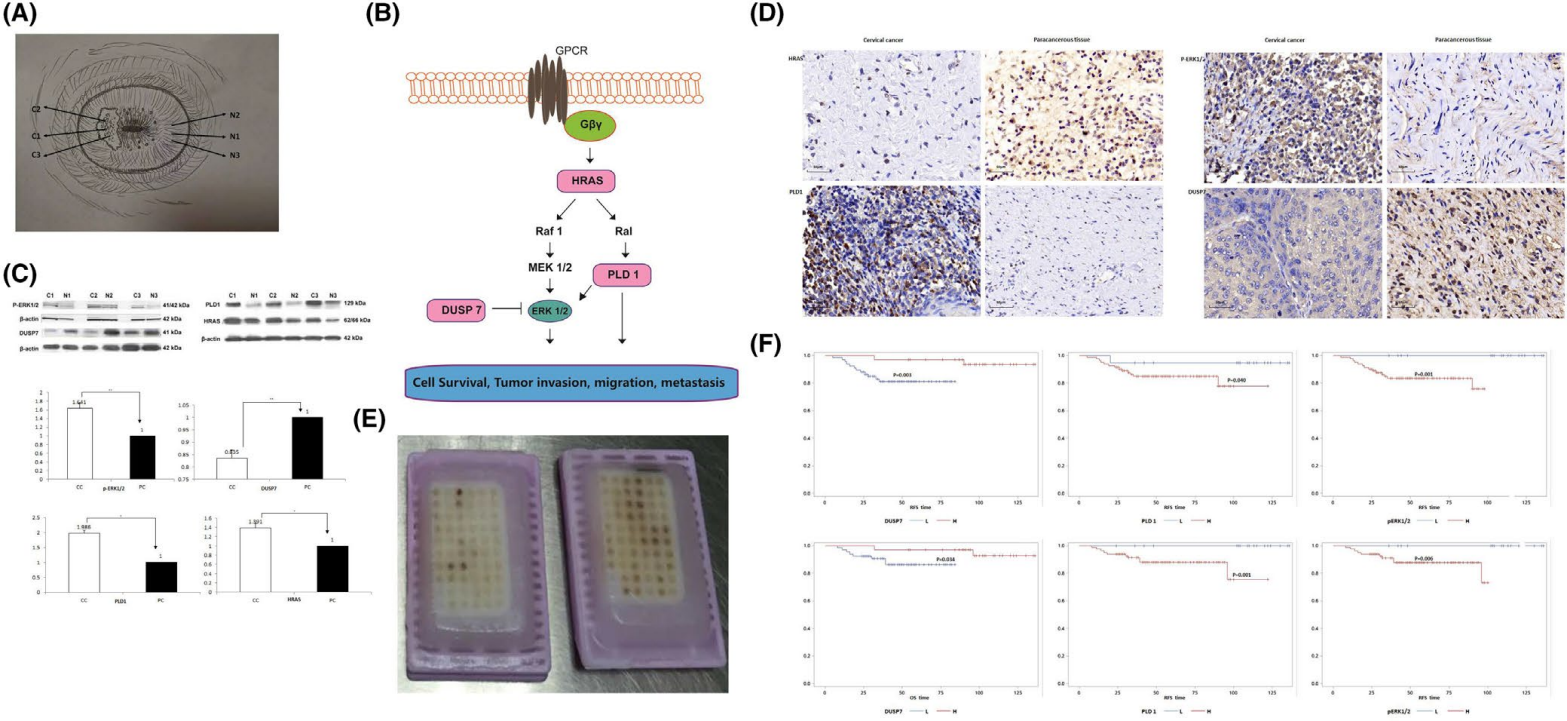
Proteomic profiles of CC and paracancerous tissue and the identification of DEPs. Sample was collected according to the ‘sandwich’ method and obtained from 3 consecutive sites of suspicious lesions (C1/C2/C3) and normal‐looking areas (N1/N2/N3). If the specimens at both ends (C2/C3, N2/N3) were consistently confirmed by pathological examination as cervical invasive carcinoma and normal cervical tissue, respectively, the middle specimens (C1 and N1) were qualified (Figure 1A). The interaction of HRAS, DUSP7, PLD1 and p‐ERK1/2 is shown in Figure 1B. WB (Figure 1C) and IHC (Figure 1D) staining analyses consistently confirmed the results of the quantitative proteomic analysis that DEPs (HRAS, P‐ERK1/2 and PLD1) levels were increased, whereas the DUSP7 level was decreased in CC tissue compared with the paired normal paracancerous tissue. A total of 102 patients’ FFPE samples were included in the TMA (Figure 1E; optical magnification*20). The IHC results from the CC TMA analysis showed that the decreased expression of DUSP7 and increased expression of PLD1 and p‐ERK1/2 were adversely related to patients’ relapse (*p* = 0.003, 0.040 and 0.001, respectively; Figure 1F) and survival (*p* = 0.034, 0.001 and 0.006, respectively). *p < 0.05, **p < 0.01

CC and paracancerous tissue samples were all collected from patients who underwent surgery at the Department of Obstetrics and Gynecology, Beijing Chao‐yang Hospital, Capital Medical University. Those who received preoperative chemotherapy or radiotherapy or had concurrent or successive primary malignancies were excluded. Patients, with extensive cancerous areas occupying the cervix, were also excluded. Samples were collected according to the ‘sandwich’ method (Figure 1A) and obtained from 3 consecutive sites of suspicious lesions (C1/C2/C3) and normal‐looking areas (N1/N2/N3). Specimens at the middle site (C1 and N1) were immediately washed 3 times with ice‐cold PBS solution and stored in liquid nitrogen. If the specimens at both ends (C2/C3 and N2/N3) were consistently confirmed by pathological examination as cervical invasive carcinoma and normal cervical tissue, respectively, the middle specimens (C1 and N1) were regarded as qualified and analysed. A total of 13 pairs of qualified specimens were analysed. The cancerous and adjacent normal tissue samples were named C1‐13 and N1‐13, respectively. Tissue collection was performed under the approval of the Beijing Chao‐yang Hospital Ethics Committee and under the patients’ informed consent.

### 2.3 Proteomic profiles of CC and paracancerous tissue and the identification of DEPs

Three pairs of *C*C and paracancerous tissue were used for proteomic testing. Samples were ground in liquid nitrogen and dissolved in PBS containing 8 M urea, 1 × protease inhibitor cocktail (Biotool, B14001) and 1 mM PMSF. After sonication for 5 min, the protein concentrations of each sample were measured with the BCA method. Each sample (100 μg) was reduced with 5 mM DTT and alkylated with 12.5 mM iodoacetamide (IAM). After being diluted with PBS to 1.5 M urea, samples were digested with trypsin at a protease/protein ratio of 1:100 overnight at 37°C. Then, Sep‐Pak columns (Waters, MA) were used to desalt the samples. According to the manufacturer's instructions, peptides from each sample were labelled with tandem mass tag (TMT) reagents (Thermo, Pierce Biotechnology). The TMT‐labelled peptides were desalted by a Sep‐Pak column.

A UPLC3000 system (Dionex, CA) with an XBridgeTM BEH300 C18 column (Waters, MA) was used to fractionate the peptides. H_2_O adjusted by ammonium hydroxide to a pH of 10 was defined as mobile phase A, and acetonitrile adjusted by ammonium hydroxide to a pH of 10 was defined as mobile phase B. Peptides were separated in a gradient manner as follows: phase B, 8%–18%, 30 min; and phase B, 18%–32%, 22 min. Peptides at forty‐eight fractions were collected, dried with a SpeedVac, combined into 12 fractions and redissolved in 0.1% formic acid.

For proteomic quantitative analysis, a 120 min gradient step elution at a flow rate of 0.250 μl/min with an EASYnLCII™ integrated nano‐HPLC system (Proxeon, Denmark) was used to separate the TMT‐labelled peptides. This system was directly interfaced with a Q Exactive mass spectrometer (Thermo Scientific). Mobile phase A consisted of only 0.1% formic acid. Mobile phase B consisted of 100% acetonitrile and 0.1% formic acid. Xcalibur 2.1.3 software was used to operate the Q Exactive mass spectrometer in the data‐dependent acquisition mode. The generated MS/MS spectra were collected with a data‐dependent acquisition method. The isolation window, dynamic exclusion time and normalized collisional energy (NCE) were set to a 2‐Da width, 60 s and 30 s, respectively. Quantification was carried out only for proteins with two or more matching unique peptides. The median value of all peptide hits belonging to a protein was defined as the protein ratio. Protein ratio variability was used to evaluate the quantitative precision. The DEPs were identified through this manner. The gene functions of the DEPs were analysed through bioinformatic analysis in combination with bibliographical information.

The DEPs with a greater likelihood of having a potential role in the pathogenesis and progression of CC were selected for validation through WB and IHC staining. The expression of the validated DEPs was detected through IHC examination of CC tissue arrays.

### 2.4 Tissue microarray (TMA) construction

A TMA was constructed based on the formalin‐fixed, paraffin‐embedded (FFPE) tumour tissues of CC patients. The inclusion criteria were as follows: (1) CC patients who underwent primary surgery at Beijing Chao‐Yang Hospital, Capital Medical University between January 2002 and December 2013. (2) Patients with cervical squamous cell carcinoma (SCC) and (3) patients who did not receive neoadjuvant chemotherapy and/or radiotherapy before surgery. Clinicopathological information, including age at diagnosis, number of pregnancies and deliveries, status of menopause, body mass index (BMI), tumour history, family history of tumour, histological type, tumour grade, FIGO stage, surgery, intraoperative and postoperative complications, postoperative radiotherapy and chemotherapy, recurrence and death, was collected from the clinical database. FFPE tumour samples were collected from the Department of Pathology Department at our hospital. Progression‐free survival (PFS) was calculated from the date of surgery to tumour recurrence. Patients who lived free from the disease at the last visit were censored. Overall survival (OS) was calculated from the date of diagnosis to patient death or the last follow‐up.

TMAs were constructed according to a method described in our previous studies ^7^, ^8^. The slides were reviewed, and the pathological diagnosis of cervical SCC of all the included patients was confirmed by two independent gynaecological pathologists who were blinded to the clinical data. Accurate locations of the tumours were marked on the FFPE samples. Two tissue cores, 1 mm in diameter, were taken from a donor block and placed in a recipient block (10 × 12 arrays) using a manual tissue array instrument. Sequential 4 µm‐thick sections were cut from the FFPE TMA blocks and mounted on blank slides.

IHC staining was performed to detect the expression level of the validated DEPs in the CC TMAs. The images of TMA slides were captured using a digital pathological section scanner (Pannoramic MIDI/P250). Pannoramic Viewer 1.15.4 software was used to display the image at 1–400 × magnification. Semiquantitative analysis was performed according to the histochemistry score (H‐score), which was calculated based on a combination of the scores for the percentage of stained cells and staining intensity. H‐score = Σ(percentage [0%–100%] × intensity ^1^, ^2^, ^3^) = (percentage of cells with weak intensity × 1) + (percentage of cells with moderate intensity × 2) + (percentage of cells with strong intensity × 3) ^7^. The validated DEPs with expression levels that were significantly associated with patients’ clinical outcomes were selected as candidate DEPs for further evaluation.

### 2.5 Production and transduction of lentiviral particles

A plasmid encoding a candidate DEP gene was inserted into the pWSLV‐08 vector, with green fluorescent protein (GFP) as the reporter gene. Lentiviral particles containing the candidate DEP gene were transfected into SIHA cells (*candidate DEP*‐SIHA). Then, the cells were cultured and amplified. GFP‐positive cells were sorted by flow cytometry (FCM). SIHA cells transfected with a negative control (NC) plasmid (NC‐SIHA) were used as a control.

Specific short hairpin RNAs (shRNAs) targeting the candidate DEP gene were designed and synthesized. Separate fragments containing different shRNAs targeting the candidate DEP gene and the scrambled shRNA sequence were each cloned into the GV248 plasmid. The GV248 plasmid and other packaging plasmids were cotransfected into HEK293T cells using Lipofectamine 2000. *Candidate DEP*‐SIHA cells were transfected with the viral particles, and the cells were collected 48 h after transfection. Cells with the viral particle containing the most effective shRNA sequence were selected and named sh‐*candidate DEP*‐SIHA.

Real‐time PCR and WB were used to validate the transfection efficiency of the candidate *DEP* at the protein and mRNA levels, respectively. Independent sample t test was used for statistical evaluation. Primers for real‐time PCR are shown in Table S3.

### 2.6 The effect of candidate DEPs on the biological function of SIHA

Based on the reports on the candidate DEPs in the literature, the following experimental procedures were performed on the *candidate DEP*‐SIHA and NC‐SIHA, as well as sh‐*candidate DEP*‐SIHA and shNC‐SIHA cells. The Cell Counting Kit‐8 (CCK‐8) assay and FCM were used to detect cell proliferation and cell cycle distribution, respectively. A colony formation assay was used to assess colony formation. The migration ability was detected through wound healing and Transwell migration assays. Transwell invasion assays were used to assess the invasion ability. Independent sample *t* test was used for statistical evaluation. Immunofluorescence assays were used to detect the expression of E‐cadherin and vimentin in the target cells. The role of the candidate DEPs in epithelial‐to‐mesenchymal transition (EMT) was also evaluated in this manner.

### 2.7 Xenograft experiments

All the following procedures were approved by the Animal Research Ethics Committee of Capital Medical University. Ten female nude mice (female BALB/c, 4 weeks of age) were randomly divided into 2 groups (5 mice per group). *Candidate DEP*‐SIHA/NC‐SIHA (or sh‐*candidate DEP*‐SIHA/shNC‐SIHA) cells were injected subcutaneously into the left or right flanks of nude mice to compare the tumour formation ability in vivo.

### 2.8 Statistical analysis

All statistical analyses were performed using Review Manager 5.3 and SPSS software version 19. A *p* value of <0.05 was considered significant. The relationship between the DEP expression and patient clinicopathological characteristics was evaluated through the Spearman rank correlation test. Kaplan‐Meier survival analysis was used to analyse the prognostic role of expression level of the DEPs. Paired sample t test was used to compare the expression level of DEP gene between CC and paracancerous tissues and tumour size between *candidate DEP*‐SIHA (or sh‐*candidate DEP*‐SIHA) and NC‐SIHA (or shNC‐SIHA) groups.

### 2.9 Ethics approval

Patient records and information were anonymized and deidentified prior to analysis; therefore, consent was not necessary. The study protocol was approved by the Ethics Committees at Beijing Chao‐Yang Hospital.

## 3 RESULTS

### 3.1 Proteomic profile of CC and paracancerous tissue and the identified DEPs

A total of 7811 proteins were identified in all samples, with less than a 1% false discovery rate (FDR) (Table S4). According to the TMT ratios (≥1.5 or ≤0.5), 129 proteins were found to be differentially expressed in all 3 pairs of samples, with a *p* value <0.05. The gene function of 97 out of the 129 DEPs, including 89 upregulated and 8 downregulated DEPs, was associated with the pathogenesis and progression of tumours (Table 1).

**TABLE 1:** The 129 DEPs with a potential role in pathogenesis and progression of tumours

Accession	Gene name	C1/ N1	C2/ N2	C3/ N3	Accession	Gene name	N1/C1	N2/C2	N3/C3
Upregulating in cancerous tissue					P01112	HRAS	1.39	3.05	1.80
O94929	ABLIM3	2.60	2.16	2.39	P35367	HRH1	1.62	3.80	2.50
K7EM38	ACTG1	1.48	1.75	1.64	P17066	HSPA6	1.50	1.57	1.64
P05062	ALDOB	5.26	1.34	1.74	C9JTH1	IL36RN	1.53	1.54	6.14
P02760	AMBP	1.34	1.94	2.05	A0A087WW43	ITIH3	1.50	1.36	1.51
C9J0G8	AOC1	2.33	2.72	1.52	Q14624‐3	ITIH4	1.77	2.06	1.59
Q06278	AOX1	1.78	1.52	1.42	A0A087WY88	JAGN1	2.69	1.88	1.42
P05090	APOD	1.35	3.35	2.06	E9PB18	KIAA1324	1.42	1.45	1.83
A7KAX9‐2	ARHGAP32	1.36	1.46	1.43	F8WCS1	MED15	2.07	2.63	1.36
Q93088	BHMT	2.69	2.10	1.98	Q14680‐3	MELK	1.89	1.66	2.12
P43251‐4	BTD	1.46	5.70	1.40	B3KW70	MFAP5	1.68	1.95	2.63
P13671	C6	1.41	1.81	1.71	H7C4E0	MGLL	1.36	1.76	1.72
P02748	C9	1.45	1.89	1.78	P12882	MYH1	1.68	2.50	5.61
P22748	CA4	2.05	1.40	1.35	A5PLL3	MYST3	1.59	1.57	3.14
Q6UXS9‐3	CASP12	2.21	1.60	2.16	Q5TD07	NQO2	1.55	1.73	1.99
Q9UK58‐5	CCNL1	1.76	1.99	1.52	P10588	NR2F6	1.87	1.71	2.57
E9PNW4	CD59	1.50	1.83	1.58	V9GY00	PBLD	2.48	1.84	1.42
O43866	CD5L	1.40	2.40	1.62	A0A087WVF8	PDE4DIP	2.93	1.51	1.62
H0YMY6	CERS4	1.52	1.62	1.58	P16234	PDGFRA	1.45	2.54	1.36
G3XAM2	CFI	1.37	2.25	1.89	**Q13393‐2**	**PLD1**	**1.39**	**1.64**	**2.09**
P10909‐4	CLU	1.56	1.87	1.78	C9JE27	RFFL	1.35	5.27	1.49
P00450	CP	1.72	2.36	1.78	O14924‐7	RGS12	1.42	1.85	1.49
P29762	CRABP1	3.37	2.00	1.71	P05109	S100A8	3.81	1.38	1.85
P54108	CRISP3	2.17	4.16	1.90	P06702	S100A9	3.98	2.39	2.12
Q9Y4D1‐2	DAAM1	1.55	2.15	1.56	H0YJH0	SAV1	2.34	1.67	1.46
Q96JQ0	DCHS1	2.28	1.77	1.49	P49908	SEPP1	1.79	17.39	1.95
Q6E0U4‐7	DMKN	1.64	2.24	5.50	G3V1Q4	SEPT7	2.03	1.84	1.76
Q08495‐3	DMTN	2.84	1.77	1.37	P01011	SERPINA3	1.64	2.70	2.00
P16444	DPEP1	1.68	1.52	2.62	P05154	SERPINA5	1.68	3.08	1.70
P27487	DPP4	2.35	2.58	2.10	P30740	SERPINB1	1.72	5.54	2.80
Q16610	ECM1	1.45	1.71	1.69	P50452‐3	SERPINB8	1.97	1.65	1.42
D6RDX7	EMB	1.94	1.51	1.62	P05546	SERPIND1	2.10	1.80	2.15
P00742	F10	2.79	1.62	1.35	H7C561	SF1	2.99	2.48	2.76
P21462	FPR1	1.75	1.59	1.49	I3L2A4	SIRT7	1.60	1.67	1.34
F5H450	FZD10	1.92	1.37	1.75	O14745	SLC9A3R1	1.50	1.88	1.50
P36959	GMPR	1.48	2.93	1.76	F8WCM9	TBX2	3.45	2.22	5.17
P62873	GNB1	1.78	1.54	1.53	H7C5E8	TF	2.90	13.55	1.70
A0A087X1J7	GPX3	1.71	1.94	2.09	Q9UNS1‐2	TIMELESS	2.16	2.45	1.76
O60565	GREM1	2.24	1.53	2.86	P28289	TMOD1	1.68	2.17	2.05
P06396	GSN	1.83	2.24	1.74	Q6ZMR5‐2	TMPRSS11A	1.73	4.03	1.96
P06396‐3	GSN	2.40	2.42	1.48	**Downregulating in cancerous tissue**				
P08263	GSTA1	3.36	1.55	1.62	**H7C4Z0**	**DUSP7 (MKPX)**	0.59	0.35	0.58
P09210	GSTA2	17.14	2.02	1.52	Q12834	CDC20	0.63	0.47	0.61
K7EK07	H3F3B	2.64	1.64	1.41	H0Y9P9	SRD5A3	0.46	0.51	0.64
Q14520‐2	HABP2	1.42	1.80	1.64	M0QXM4	SLC1A5	0.33	0.26	0.53
P16403	HIST1H1C	8.83	1.54	1.42	P33552	CKS2	0.65	0.62	0.26
Q30167	HLA‐DRB1	1.73	1.67	1.65	Q9UHB6‐4	LIMA1	0.64	0.61	0.63
Q5JSK7	HMGN5	2.07	2.34	1.59	Q15155	NOMO1	0.62	0.47	0.65
P00739	HPR	1.52	2.09	1.59	P08254	MMP3	0.55	0.38	0.46

Note

TMT:tandem mass tags;C1, C2, C3:cervical cancer tissue;N1、N2、N3:paired paracancerous tissue.

To investigate the functions of the DEP‐related genes in signal transduction in tumour cells, Kyoto Encyclopedia of Genes and Genomes (KEGG) pathway analysis was performed. Three DEPs, HRAS, DUSP7 and PLD1, are RAS pathway components. ERK, also a RAS pathway component, was not included in the DEPs. Total ERK is not necessarily highly expressed in human malignancies ^9^; however, persistent ERK1/2 activation through phosphorylation ultimately promotes cell proliferation and malignant transformation ^10^. The interaction of HRAS, DUSP7, PLD1 and phosphorylated ERK1/2 (p‐ERK1/2) is illustrated in Figure 1B. These four genes were thus selected for further verification. WB and IHC staining analyses confirmed the results of the quantitative proteomic analysis that the levels of HRAS, PLD1 and p‐ERK1/2 were increased, whereas the DUSP7 level was decreased in CC tissue compared with the paired normal paracancerous tissue (Figure 1C,D).

### 3.2 TMA construction and patients’ clinicopathological information

During the study period, a total of 102 patients’ FFPE samples were included in the TMA (Table 2, Figure 1E). The mean age of patients at diagnosis was 44.7 (range: 24–78) years old. The mean diameter of the tumours was 2.9 (range: 0.3–5.5) cm, including 38 (37.3%) cases of tumours with a tumour size <2 cm. Tumours were grade in I in 16 cases and grade Ⅱ+Ⅲ in 86 cases. Lymph‐vascular space invasion (LVSI) was detected in approximately one‐fourth of patients. Uterine isthmus involvement was identified in 4 (3.9%) cases. Parametrial invasion and vaginal invasion were identified in 11 and 12 (11.8%) cases, respectively. Positive lymph node involvement was identified in 18 (17.7%) cases. Postoperative adjuvant radiotherapy was administered in 68 (6.7%) cases. The mean follow‐up period was 64.6 (range: 8–136) months, during which time 14 (13.7%) women relapsed after a mean relapse interval of 26.1 (range: 5–90) months. At the last contact, 10 patients died of the disease. The 5 years relapse‐free survival rate was 86.9%, and the 5 years overall survival rate was 90.5%.

**TABLE 2:** The clinicopathological features of 102 patients with cervical cancer

Parameters	Number of patients	%	Parameters	Number of patients	%
Age (mean; range)	44.7 ± 11.1; 24–78		Parametrial invasion		
≤45	57	55.9	+	11	10.8
>45	45	44.1	−	91	89.2
Tumour size^a^(mean; range)	2.9 ± 1.2; 0.3–5.5		LNM^c^		
≤2 cm	38	37.3	+	18	17.6
>2 cm	64	62.7	−	84	82.4
FIGO stage			Positive margin		
Ia2+Ib1	49	48.0	+	4	3.9
Ib2+IIa	53	52.0	−		
Grade			Adjuvant radiotherapy		
1	16	15.7	+	68	66.7
2+3	86	84.3	−	34	33.3
LVSI^b^			Follow‐up time (month; range)	64.6 ± 32.6;8–136	
+	26	25.5	Recurrence	14	13.7
−	76	74.5	Follow‐up period (month; range)	26.1 ± 20.1; (5–90)	
Uterine isthmus involvement			Status at the last contact		
+	4	3.9	NED^d^	88	86.3
−	98	96.1	AWD^e^	4	3.9
Vaginal invasion			DOD^f^	10	9.8
+	12	11.8	5‐RFS	86.9	
−	90	88.2	5‐OS	90.5	

^a^ Clinically measurable tumours only.

^b^ Lymphovascular space involvement.

^c^ Lymph node metastasis.

^d^ No evidence of disease.

^e^ Alive with disease.

^f^ Dead of disease.

^g^ Relapse‐free survival.

^f^ Overall survival.

### 3.3 The expression of DUSP7, PLD1 and p‐Erk1/2 in CC TMA

The IHC results from the CC TMA analysis showed that the decreased expression of DUSP7 (*p* = 0.045 and 0.044, respectively) and increased expression of PLD1 (*p* = 0.046 and 0.028, respectively) were significantly associated with a tumour size >2 cm and parametrial infiltration (Table 3). The Pearson correlation coefficients for the expression of PLD1 and p‐Erk1/2 vs. DUSP7 in the CC TMA were −0.964 (95% CI: −0.986, −0.431; *p* = 0.005) and −0.545 (95% CI: −0.856, −0.241; *p* = 0.002), respectively. The expression of PLD1 and p‐Erk1/2 was both significantly negatively correlated with that of DUSP7. In addition, the decreased expression of DUSP7 and increased expression of PLD1 and p‐ERK1/2 were adversely related to patients’ relapse (*p* = 0.003, 0.040 and 0.001, respectively; Figure 1F) and survival (*p* = 0.034, 0.001 and 0.006, respectively). DUSP7 was selected as the candidate gene and was evaluated in further experiments.

**TABLE 3:** The expression of DUSP7, PLD1 and p‐Erk1/2 in the cervical cancer tissue microarray

Parameter	HRAS		DUSP7		PLD1		p‐Erk1/2	
	TISS^a^	*p* value^b^	TISS^a^	*p* value^b^	TISS^a^	*p* value^b^	TISS^a^	*p* value^b^
Age								
≤45	5.26 ± 3.34	0.894	3.28 ± 1.33	0.878	5.37 ± 1.07	0.921	6.44 ± 2.41	0.465
>45	6.02 ± 3.79		3.81 ± 1.54		5.66 ± 1.15		7.16 ± 1.25	
Tumour size								
**≤**2 cm	5.25 ± 2.56	0.065	4.35 ± 2.01	0.045	5.15 ± 0.78	**0**.**044**	6.43 ± 3.12	0.069
>2 cm	7.11 ± 1.47		2.72 ± 1.68		5.97 ± 2.47		7.52 ± 2.08	
Stage								
Ia2+Ib1	4.93 ± 2.112	0.168	3.36 ± 1.38	**0**.**004**	5.07 ± 1.11	**<0**.**001**	6.29 ± 1.66	**0.007**
Ib2+IIa	6.72 ± 1.3		1.99 ± 1.77		6.03 ± 2.05		8.27 ± 3.08	
Grade								
1	5.42 ± 3.21	0.661	3.33 ± 1.57	0.804	5.35 ± 1.07	0.094	6.43 ± 1.88	0.558
2+3	6.23 ± 2.90		2.32 ± 1.90		5.72 ± 0.78		7.35 ± 2.22	
LVSI								
+	6.12 ± 1.77	0.988	2.09 ± 2.37	0.179	5.84 ± 2.16	0.065	7.68 ± 3.12	0.102
−	5.72 ± 2.31		3.07 ± 1.31		5.29 ± 2.33		6.44 ± 4.01	
Uterus isthmus invasion								
+	6.08 ± 1.32	0.878	2.15 ± 1.04	0.243	5.64 ± 1.28	0.153	7.45 ± 2.58	0.754
−	5.75 ± 1.62		3.22 ± 1.89		5.49 ± 1.28		6.57 ± 3.67	
Vaginal invasion								
+	6.33 ± 2.41	0.761	1.76 ± 1.98	0.084	5.35 ± 1.34	0.092	7.92 ± 2.22	0.098
−	5.75 ± 1.2		3.74 ± 1.43		5.76 ± 1.78		6.36 ± 1.09	
Parametrial invasion								
+	7.92 ± 1.21	0.068	4.25 ± 1.08	**0**.**046**	5.88 ± 1.23	**0**.**028**	6.16 ± 3.08	0.665
−	6.14 ± 2.03		2.32 ± 1.48		5.29 ± 1.15		5.06 ± 1.44	
LNM								
+	7.96 ± 2.16	0.075	1.06 ± 1.22	**<0**.**001**	6.98 ± 3.31	**<0**.**001**	9.12 ± 1.81	**<0.001**
−	5.35 ± 1.14		3.33 ± 1.41		5.11 ± 1.83		6.33 ± 1.36	

^a^ Specific Index Total Cellular Immunostaining Scoring.

^b^ Wilcoxon signed‐rank test.

### 3.4 Establishment of stable DUSP7 knockdown and overexpression in SIHA cells

**FIGURE 2:**
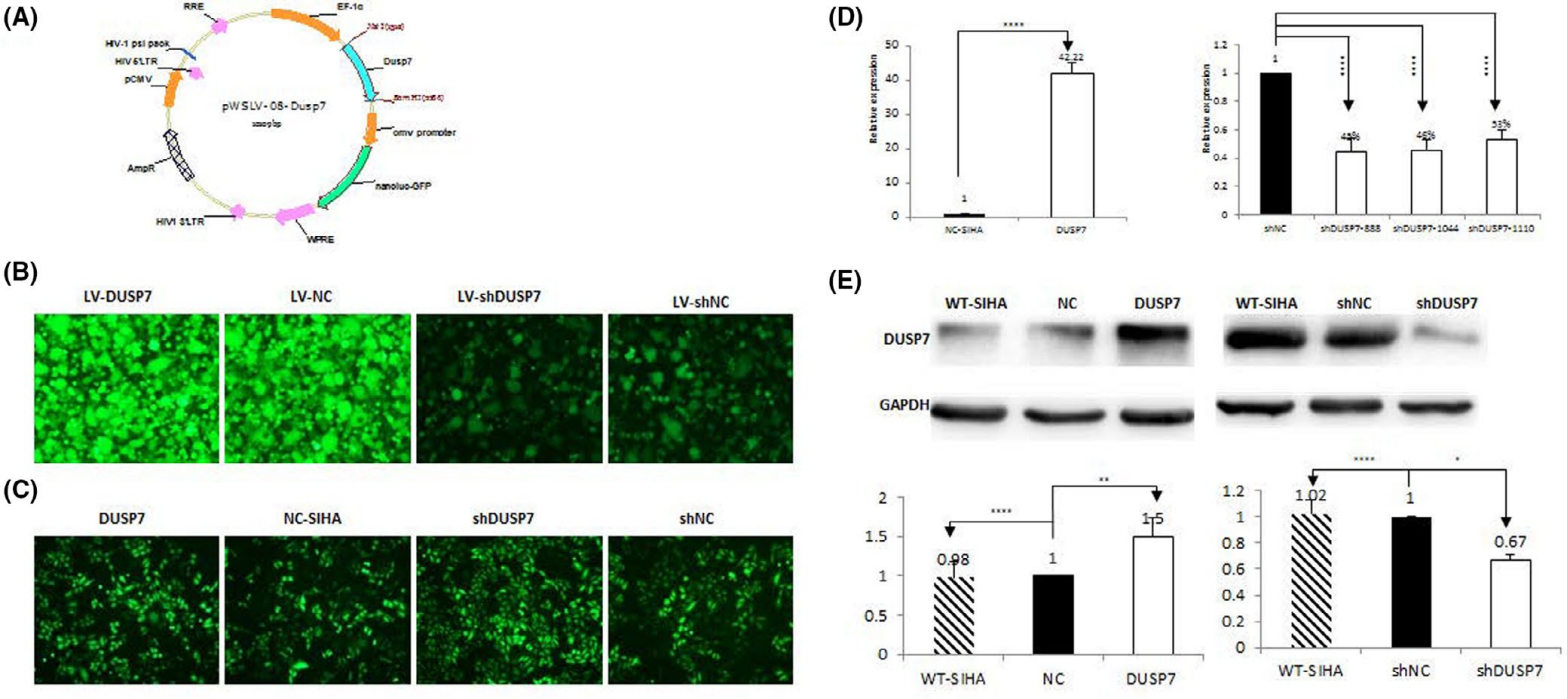
Establishment of DUSP7 knockdown and stable overexpression in SIHA cells. Stable DUSP7‐overexpressing cell lines were established by transducing SIHA cells with Lv‐DUSP7 and were named *DUSP7*‐SIHA cells (Figure 2A,B; optical magnification*20). The qRT‐PCR results showed that the DUSP7 mRNA level in *DUSP7*‐*SIHA* cells was significantly higher than that in NC‐SIHA cells (42.52 vs. 1, *p* < 0.0001; Figure 2C). Three specific shRNAs targeting DUSP7 were designed and synthesized. The qRT‐PCR results showed that the silencing efficiencies of these shRNAs were 55%, 54% and 47% (all *p* < 0.0001) when shNC was used as a reference. Cells infected with the most effective (55%) shRNA sequence (GCAUCAAGUAUAUCCUCAATT) were named shDUSP7‐SIHA and used for subsequent experiments. The WB results indicated that the DUSP7 expression level in *Dusp7*‐SIHA cells was significantly higher than that in NC‐SIHA and wild‐type SIHA cells. In contrast, DUSP7 expression was significantly downregulated in shDUSP7‐SIHA cells compared with *Dusp7*‐SIHA and wild‐type SIHA cells (Figure 2D,E). *** *p* < 0.001, **** *p* < 0.0001

The plasmid of DUSP7 gene was constructed into pWSLV‐08 vector and renamed as pWSLV‐08‐DUSP7 (Figure 2A). Primer information was shown as follows. The NotI‐Dusp7‐Fp was 'AAGGAAAAAAGCGGCCGCGCCACCATGTTGCGCCGCCTGCGCAA'. The amHI‐Dusp7‐RP was 'CGCGGATCCTCACGTGGACTCCAGCGTAT'. The dusp7‐FP was 'AAGTTATATTAAGGGTTCCA'. The dusp7‐Mfcexu was 'CTGTGCCATCCAGCCAACCA'. The Ef1a‐sq was 'CACTTGATGTAATTCTCCTTGGAAT'. Stable DUSP7‐overexpressing cell lines were established by transducing SIHA cells with Lv‐DUSP7 and were named *DUSP7*‐SIHA cells (Figure 2B,C). The qRT‐PCR results showed that the DUSP7 mRNA level in *DUSP7*‐*SIHA* cells was significantly higher than that in NC‐SIHA cells (42.52 vs. 1, *p* < 0.0001; Figure 2D). Three specific shRNAs targeting DUSP7 were designed and synthesized. The qRT‐PCR results showed that the silencing efficiencies of these shRNAs were 55%, 54% and 47% (all *p* < 0.0001) when shNC was used as a reference. Cells infected with the most effective (55%) shRNA sequence (GCAUCAAGUAUAUCCUCAATT) were named shDUSP7‐SIHA and used for subsequent experiments. The WB results indicated that the DUSP7 expression level in *Dusp7*‐SIHA cells was significantly higher than that in NC‐SIHA, which was significantly downregulated in shDUSP7‐SIHA cells compared with that in shNC‐SIHA cells. DUSP7 expression in NC‐SIHA and shNC‐SIHA both was similar to that in wild‐type SIHA cells (Figure 2E).

### 3.5 The effect of DUSP7 on the biological function of SIHA cells

**FIGURE 3:**
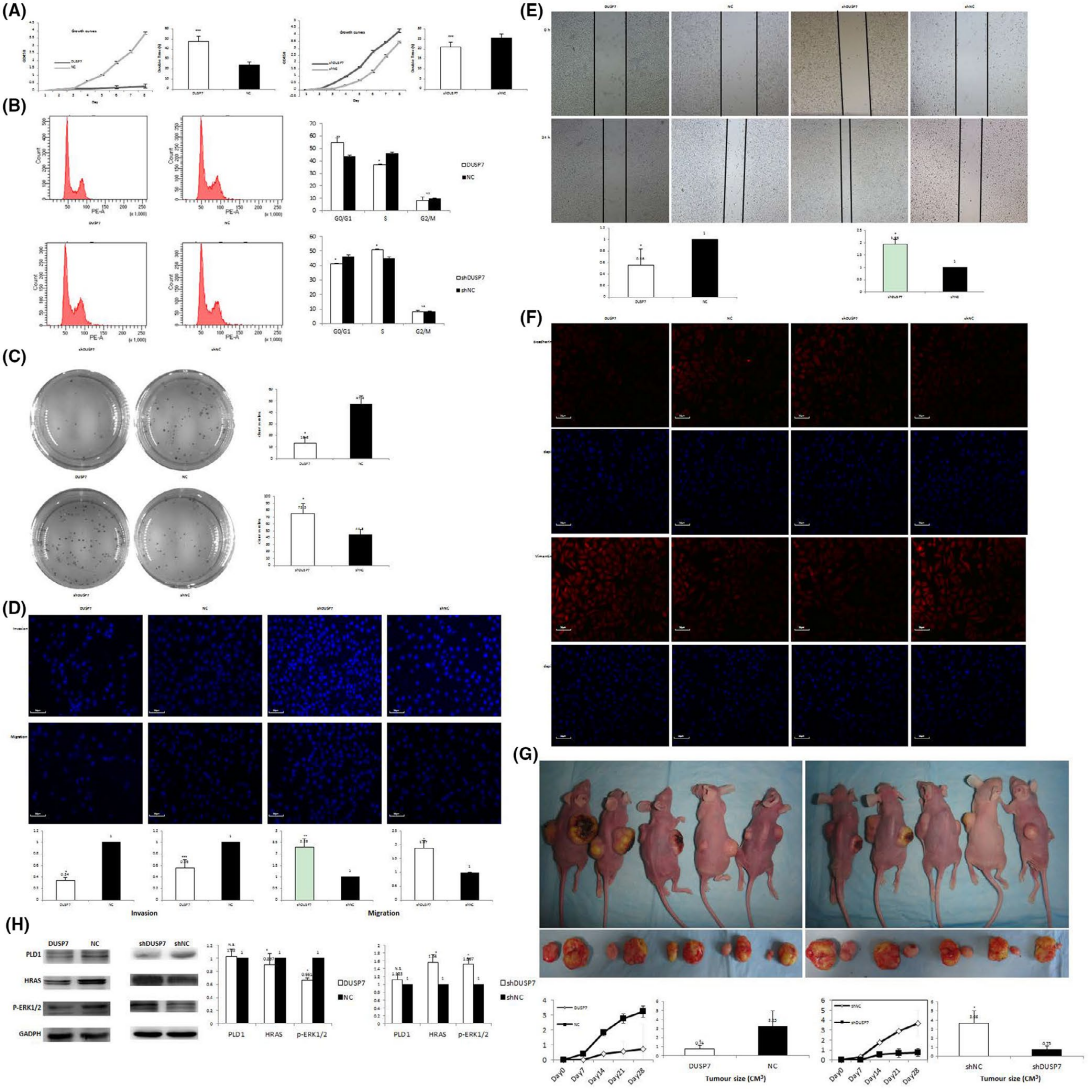
The effect of DUSP7 on the biological function of SIHA cells. The CCK‐8 assay growth curves showed that DUSP7‐SIHA cells proliferated significantly slower than NC‐SIHA cells, based on a clear delay in the doubling time (47.72 ± 1.14 h vs. 23.99 ± 0.47 h, *p* = 0.0001; Figure 3A). Cell cycle analysis indicated that the DUSP7‐SIHA cells displayed a concomitant decrease in the percentage of cells in S phase (37.71 ± 0.53% vs. 46.96 ± 0.59%, *p* < 0.0001) and a significant increase in the percentage of cells in G0/G1 phase (52.50 ± 3.49% vs. 44.04 ± 0.71%, *p* = 0.0473; Figure 3B). Colony formation assays showed that the number of colonies formed by DUSP7‐SIHA cells was significantly less than that formed by NC‐SIHA cells (44.67 ± 9.0 vs. 75.33 ± 14.47, *p* = 0.0121; Figure 3C). In the Matrigel invasion/migration assay, DUSP7‐SIHA cells demonstrated a significantly weaker ability to invade (0.34 ± 0.05 vs.1, *p* = 0.0207; Figure 3D; optical magnification*20) and migrate (0.56 ± 0.14 vs.1, *p* = 0.0059) through the membrane than control cells. Wound‐healing assays showed that the migration area of DUSP7‐SIHA cells was significantly smaller than that of NC‐SIHA cells (0.55 ± 0.03 vs.1, *p* = 0.049; Figure 3E). E‐cadherin expression was significantly increased, but vimentin expression was significantly reduced in DUSP7‐SIHA cells (Figure 3F). In contrast, the results of CCK‐8 assay growth curves indicated that the doubling time of shDUSP7‐SIHA cells was significantly shorter than that of shNC‐SIHA cells (49.12 ± 1.14 h vs. 64.14 ± 0.47 h, *p* = 0.0001; Figure 3A). Cell cycle analysis indicated that shDUSP7‐SIHA cells displayed a concomitant increase in the percentage of cells in S phase (49.54 ± 1.53% vs. 46.4 ± 0.97%, *p* = 0.019) and a significant decrease in the percentage of cells in G0/G1 phase (41.8 ± 0.38% vs. 45.2 ± 0.80%, *p* = 0.020; Figure 3B). Colony formation assays showed that the number of colonies formed by shDUSP7‐SIHA cells was significantly greater than that formed by shNC‐SIHA cells (13.33 ± 3.4 vs. 30.33 ± 16.50, *p* = 0.049; Figure 3C). In the Matrigel invasion/migration assay, DUSP7‐SIHA cells demonstrated a significantly weaker ability to invade (2.29 ± 0.38 vs. 1, *p* = 0.0007; Figure 3D) and migrate (1.87 ± 0.28 vs.1, *p* = 0.0426) through the membrane than control cells (*p* = 0.0207 and 0.0059, respectively; Figure 3D). Wound‐healing assays showed that the migration area of shDUSP7‐SIHA cells was larger than that of shNC‐SIHA cells (1.95 ± 0.19 vs.1, *p* = 0.0313; Figure 3E). E‐cadherin expression was significantly reduced, while vimentin expression was increased, in shDUSP7‐SIHA cells (Figure 3F; optical magnification*20). After subcutaneous injection, DUSP7‐SIHA tumours were observed much later than NC‐SIHA tumours (13 ± 4 vs 6 ± 1 days; *p* = 0.0122; Figure 3G). On the study end date, the DUSP7‐SIHA tumours were significantly smaller than the NC‐SIHA tumours (0.74 ± 0.38 vs 3.25 ± 1.68 cm^3^; *p* = 0.0183). In contrast, the shDUSP7‐SIHA tumours were observed much earlier than the shNC‐SIHA tumours (7 ± 1 vs 12 ± 2 days; *p* = 0.0303). On the study end date, the shDUSP7‐SIHA tumours were significantly larger than the shNC‐SIHA tumours (3.66 ± 1.33 vs 0.75 ± 0.41 cm^3^; *p* = 0.0201). The expression of HRAS and p‐ERK1/2 was decreased in DUSP7‐SIHA cells compared with NC‐SIHA cells (*p* = 0.0003 and 0.0026, respectively; Figure 3H). In contrast, the expression of HRAS and p‐ERK1/2 was significantly upregulated in shDUSP7‐SIHA cells compared with control cells (*p* = 0.034 and 0.0026, respectively). The difference in the expression level of PLD1 between the two cell groups was not statistically significant (*p* = 0.0947 and 0.307, respectively)

The CCK‐8 assay growth curves showed that DUSP7‐SIHA cells proliferated significantly slower than NC‐SIHA cells, based on a clear delay in the doubling time (47.72 ± 1.14 h vs. 23.99 ± 0.47 h, *p* = 0.0001; Figure 3A). Cell cycle analysis indicated that the DUSP7‐SIHA cells displayed a concomitant decrease in the percentage of cells in S phase (37.71 ± 0.53% vs. 46.96 ± 0.59%, *p* < 0.0001) and a significant increase in the percentage of cells in G0/G1 phase (52.50 ± 3.49% vs. 44.04 ± 0.71%, *p* = 0.0473), suggesting that inhibited proliferation of DUSP7‐SIHA cells may be due to the arrest of DNA synthesis (Figure 3B). Colony formation assays showed that the number of colonies formed by DUSP7‐SIHA cells was significantly less than that formed by NC‐SIHA cells (44.67 ± 9.0 vs. 75.33 ± 14.47, *p* = 0.0121; Figure 3C). In the Matrigel invasion/migration assay, DUSP7‐SIHA cells demonstrated a significantly weaker ability to invade (0.34 ± 0.05 vs.1, *p* = 0.0207; Figure 3D) and migrate (0.56 ± 0.14 vs.1, *p* = 0.0059) through the membrane than control cells. Wound‐healing assays showed that the migration area of DUSP7‐SIHA cells was significantly smaller than that of NC‐SIHA cells (0.55 ± 0.03 vs.1, *p* = 0.049; Figure 3E). Additionally, E‐cadherin expression was significantly increased but vimentin expression was significantly reduced in DUSP7‐SIHA cells, which demonstrated that overexpression of DUSP7 has a potential role in inhibiting the EMT process of SIHA cells (Figure 3F).

In contrast, the CCK‐8 assay growth curves showed that the doubling time of shDUSP7‐SIHA cells was significantly shorter than that of shNC‐SIHA cells (49.12 ± 1.14 h vs. 64.14 ± 0.47 h, *p* = 0.0001; Figure 3A). Cell cycle analysis indicated that shDUSP7‐SIHA cells displayed a concomitant increase in the percentage of cells in S phase (49.54 ± 1.53% vs. 46.4 ± 0.97%, *p* = 0.019) and a significant decrease in the percentage of cells in G0/G1 phase (41.8 ± 0.38% vs. 45.2 ± 0.80%, *p* = 0.020; Figure 3B). Colony formation assays showed that the number of colonies formed by siDUSP7‐SIHA cells was significantly greater than that formed by shNC‐SIHA cells (13.33 ± 3.4 vs. 30.33 ± 16.50, *p* = 0.049; Figure 3C). In the Matrigel invasion/migration assay, shDUSP7‐SIHA cells demonstrated a greater ability to invade (2.29 ± 0.38 vs. 1, *p* = 0.0007; Figure 3D) and migrate (1.87 ± 0.28 vs.1, *p* = 0.0426, respectively) through the membrane than control cells. Wound‐healing assays showed that the migration area of shDUSP7‐SIHA cells was larger than that of shNC‐SIHA cells (1.95 ± 0.19 vs.1, *p* = 0.0313; Figure 3E). Additionally, E‐cadherin expression was significantly reduced, while vimentin expression was increased, in shDUSP7‐SIHA cells (Figure 3F).

### 3.6 The role of DUSP7 in tumour formation in vivo

After subcutaneous injection, DUSP7‐SIHA tumours were observed much later than NC‐SIHA tumours (13 ± 4 vs 6 ± 1 days; *p* = 0.0122; Figure 3G). On the study end date, the DUSP7‐SIHA tumours were significantly smaller than NC‐SIHA tumours (0.74 ± 0.38 vs 3.25 ± 1.68 cm^3^; *p* = 0.0183). In contrast, shDUSP7‐SIHA tumours were observed much earlier than shNC‐SIHA tumours (7 ± 1 vs 12 ± 2 days; *p* = 0.0303). On the study end date, the shDUSP7‐SIHA tumours were significantly larger than the shNC‐SIHA tumours (3.66 ± 1.33 vs 0.75 ± 0.41 cm^3^; *p* = 0.0201).

### 3.7 Correlation between DUSP7 expression and the Ras pathway

The expression of HRAS and p‐ERK1/2 was decreased in DUSP7‐SIHA cells compared with NC‐SIHA cells (*p* = 0.0003 and 0.0026, respectively; Figure 3H). In contrast, the expression of HRAS and p‐ERK1/2 was significantly upregulated in shDUSP7‐SIHA cells compared with control cells (*p* = 0.034 and 0.0026, respectively). The difference in the expression level of PLD1 between the two cell groups was not statistically significant (*p* = 0.0947 and 0.307, respectively).

## 4 DISCUSSION

Proteomics, as the leading technology in the postgenomic era, plays an important role in screening diagnostic and therapeutic markers for many human malignancies ^11^. In this analysis, the ‘sandwich’ sampling method was adopted to ensure the accuracy of the histopathological results of the sampling. Cervical SCC and paired adjacent cervical tissues with the same genetic backgrounds and high comparability were used as the experimental and control groups, respectively. DEPs identified in this way could relatively objectively reflect the process of tumorigenesis and progression of CC. In this study, a total of 7811 proteins were identified through quantitative proteomics. The number of proteins identified in this study was large, and the quality of proteome detection was satisfactory.

In this study, 97 out of 129 DEPs were found to be related to tumorigenesis and the development of human malignancies, including 88 upregulated and 8 downregulated proteins. KEGG pathway analysis showed that 3 DEPs—HRAS, DUSP7 and PLD1—are RAS pathway components. WB and IHC staining analyses consistently confirmed the results of the quantitative proteomic analysis indicating that the HRAS, P‐ERK1/2 and PLD1 levels were increased while the DUSP7 level was decreased in CC tissue compared with paired normal paracancerous tissue. The RAS/RAF/ERK1/2 signalling pathway, which involves members of the mitogen‐activated protein kinase (MAPK) family, is pivotal in cell signalling networks ^12^. The RAS/RAF/MEK/ERK pathway can trigger a series of cascade reactions, namely, protein phosphorylation, amplification of upstream molecular signals and transduction of the signal into the nucleus, thus activating transcription, promoting gene expression and stimulating infinite cells ^12^. RAS has 4 isoforms: HRAS, NRAS, KRAS4A and KRAS4B. HRAS gene overexpression can specifically activate RAF/MEK/ERK and accelerate the G1/S phase transformation of CC cells ^13^. MEK/ERK activation is associated with CC cell resistance to cisplatin ^14^. However, when a large number of phosphorylated ERKs accumulate in the nucleus, the use of the MEK‐specific blocker U0126 cannot reverse the resistance response of ovarian cancer cells to cisplatin ^15^. Exploring the regulatory mechanism of ERK1/2 and reversing its phosphorylation are future areas of focus for oncologists.

Based on our data, the decreased expression of DUSP7 and increased expression of PLD1 were significantly associated with a tumour size >2 cm and parametrial infiltration. In addition, increased expression of p‐ERK1/2 and PLD1 and decreased expression of DUSP7 in the CC tissue array were adversely related to patient relapse and survival. These results indicate that both DUSP7 and PLD1 have important regulatory roles in the tumorigenic effect of p‐ERK1/2 in CC. DUSP7 is a member of the dual‐specificity phosphatase (DUSP) family. As a negative regulator of MAPK, DUSPs are involved in cell growth, differentiation, proliferation, migration, apoptosis and tumour formation ^16^. Many studies have confirmed that DUSPs are related to tumorigenesis and development. DUSP4 is considered a candidate tumour suppressor gene, and its deletion is related to the occurrence of breast cancer, rectal cancer, thyroid cancer and other tumours ^17^, ^18^. DUSP6 is expressed at low levels in the tumour tissues of many human malignancies, including ovarian cancer and endometrial cancer ^19^, ^20^. DUSP1 plays different roles in human tumorigenesis; specifically, it acts as a cancer‐promoting factor in lung cancer and leukaemia ^21^, ^22^, and as a tumour suppressor in head and neck SCC, prostate cancer and urothelial bladder cancer ^23^, ^24^.

The DUSP7 gene is located on human chromosome 3p21 ^25^. DUSP7 has a MAP kinase‐binding domain/kinase‐acting region that can specifically bind to p‐ERK1/2, thus leading to its dephosphorylation. In this manner, DUSP7 can promote the meiosis of oocytes ^26^, the loss of pluripotency in embryonic stem cells ^14^ and the differentiation of T cells ^27^. Cooperation with DUSP6 and DUSP9 promotes the development of the middle ear and outer ear in mice ^28^. However, the role of DUSP7 in the development of human tumours is still poorly understood and controversial. In 2003, Nissenbaum and his colleagues ^29^, ^30^, ^31^ found that DUSP7 was upregulated in peripheral blood mononuclear cells and bone marrow in patients with acute leukaemia. However, a more recent study demonstrated that DUSP7 is a tumour suppressor gene. DUSP7 deletion has been identified in a variety of human mesothelioma cells ^32^ and tumour tissues ^33^. DUSP7 activation can effectively block the cell cycle of (human or mouse) BRCA2‐deficient cells and significantly inhibit proliferative activity ^34^. Ham et al. ^35^ demonstrated that constitutive DUPS6 and DUSP7 expression is inversely related to the expression of inducible DUSPs and the phosphorylation of ERK1/2 in lipopolysaccharide (LPS)‐stimulated microglia. DUSP7 downregulation is associated with poor survival in patients with breast cancer ^36^. However, the relationship between DUSP7 and tumorigenesis of CC has not been reported in the literature. In this analysis, high expression of DUSP7 significantly inhibited the proliferation, invasion and migration ability and EMT process of SIHA cervical cancer cells. The tumorigenesis ability of SIHA cells in nude mice was also inhibited in this manner. In contrast, when DUSP7 expression was decreased, the anchorage‐independent growth of SIHA cells was significantly increased. In addition, when DUSP7 mRNA levels were up‐ or downregulated, the expression of HRAS and p‐ERK1/2 in SIHA cells was significantly reduced or increased, respectively. ERK inactivation arrested cells at late G1 phase and did not prevent cells from entering S phase and from transitioning from G2 to M phase ^37^, ^38^. Persistent ERK 1/2 phosphorylation is the key for signal transmission from surface receptors to the nucleus, and its continuous activation ultimately promotes cell proliferation and malignant transformation ^39^. These data indicated that the biological function of DUSP7 is possibly achieved through dephosphorylation of ERK1/2 and inactivation of the RAS pathway. The expression level of PLD1 is not affected by the upregulation or downregulation of DUSP7, and the effect of PLD1 on the progression of cervical cancer is not dependent on the activity status of DUSP7. The related mechanism remains unclear and warrants additional research.

### 4.1 Strengths and Limitations

This study has several strengths compared with other contemporary studies. First, CC and paracancerous tissue samples used for proteomics testing were collected according to the ‘sandwich’ method and underwent relatively strict pathological examination. DEPs identified in this manner accurately reflect the tumorigenesis of CC. Moreover, this study provides the first evidence that DUSP7 inhibits CC progression by inactivating the RAS pathway. However, some important limitations also exist in this study. First, IHC staining for DUSP7 in human CC tissues must be interpreted carefully due to the drawbacks of TMAs. TMAs including the limited tissues collected in the present study may not be representative of the whole tumour. In addition, due to the limitations of basic research, the effects of DUSP7 and its correlation with the RAS pathway should be verified in the future.

## 5 CONCLUSIONS

DUSP7 is decreased in cervical cancer tissues compared with normal tissues. Increasing or decreasing DUSP7 expression was found to significantly reduce or enhance the anchorage‐independent growth of SIHA cells, respectively. The biological function of DUSP7 is possibly achieved through dephosphorylation of ERK1/2 and inactivation of the RAS pathway. Upregulating the expression of DUSP7 may be useful for the prevention or treatment of CC.

## CONFLICT OF INTERESTS

The authors have no conflicts of interest to declare.

## AUTHOR CONTRIBUTION

**Huimin Bai:** Conceptualization (lead); Data curation (lead); Formal analysis (lead); Funding acquisition (lead); Investigation (lead); Methodology (lead); Project administration (lead); Resources (lead); Software (lead); Supervision (lead); Validation (lead); Visualization (lead); Writing‐original draft (lead); Writing‐review & editing (lead). **Meiying Song:** Conceptualization (lead); Data curation (lead); Formal analysis (lead); Investigation (lead); Methodology (lead); Project administration (lead); Supervision (lead); Visualization (lead); Writing‐review & editing (lead). **Ruili Jiao:** Conceptualization (equal); Data curation (equal); Investigation (equal); Software (equal); Validation (equal); Visualization (equal). **Weihua Li:** Funding acquisition (equal); Investigation (equal); Methodology (equal); Software (equal). **Jing Zhao:** Methodology (equal); Project administration (equal); Software (equal); Validation (equal). **Meizhu Xiao:** Data curation (equal); Funding acquisition (equal); Investigation (equal); Methodology (equal); Validation (equal). **Mulan Jin:** Formal analysis (equal); Investigation (equal); Methodology (equal); Project administration (equal); Software (equal). **Haiteng Deng:** Data curation (lead); Supervision (lead); Validation (lead); Visualization (lead); Writing‐original draft (lead). **Zhenyu Zhang:** Conceptualization (lead); Data curation (lead); Formal analysis (lead); Investigation (lead); Supervision (lead); Visualization (lead); Writing‐original draft (lead); Writing‐review & editing (lead).

## ETHICAL APPROVAL

The collection of CC and paired paracancerous tissue samples and the construction of CC TMA were under the approval of Beijing Chao‐yang Hospital Ethics Committee and under the patients’ informed consent.

## CONSENT FOR PUBLICATION

All the authors have reviewed the manuscript and the related files and consented to its publication.

## Supporting information

Table S1

Table S2

Table S3

Table S4

## Data Availability

The data sets supporting the results of this article are included within the article and its additional files.
