# Supplementary material for: DUSP7 inhibits cervical cancer progression by inactivating the RAS pathway
Source: J Cell Mol Med. 2021 Aug 26;25(19):9306–18. doi: 10.1111/jcmm.16865 (PMC8500958; doi:10.1111/jcmm.16865)
Supplement: Supplementary file 1 — Table S1 [file JCMM-25-9306-s001.docx]

Table S1 The main materials used in this work.

| **Materials** | | **Supplier** |
| --- | --- | --- |
| Human cervical cancer cell line SIHA | | Cell Support Center of Institute of Basic Medical Sciences, Chinese Academy of Medical Sciences. |
| Cell culture | | |
|  | RPMI-1640 | HyClone Laboratories, Inc. |
|  | Fetal bovine serum (FBS) |  |
|  | Trypsin-EDTA |  |
|  | Penicillin streptomycin |  |
|  | Culture dishes for all levels | Corning Inc, USA |
| Lipofectamine^TM^2000 kit | | Invitrogen, USA |
| CCK 8 reagent | | DOJINDO,Japan |
| 3-(4,5-Dimethyl-2-thiazolyl)-2,5-diphenyl-2-H-tetrazolium bromide (MTT) | | AmrescoInc, USA |
| Cell cycle detection kit | | Haiji, China |
| BD Matrigel TM Basement Matrix | | BD Biosciences, USA |
| Annexin V-FITC/PI Apoptosis Detection Kit | | BD Biosciences, USA |
| Female BALB/c nude mice | | Institute of Laboratory Animals of the Chinese Academy of Medical Sciences and maintained under pathogen-free conditions (CAMS &PUMC)* |
| Instruments | | |
|  | LC-MS (Q-Exactive) | Thermo Scientific |
|  | Pathological tissue embedding machine (BM-II) | Anhui, China |
|  | Baking sheet machine (CS-V) | Hongye, China |
|  | ELISA reader | Multiskan MS, Labsystem, Finland |
|  | Flow cytometer | Beckman Coulter, USA |
|  | An Applied Biosystems 7500 Real-time PCR system | Applied Biosystems, USA |

Note: * The institute is certified by the Association for Assessment and Accreditation of Laboratory Animal Care (AAALAC, USA).
