# Supplementary material for: DUSP7 inhibits cervical cancer progression by inactivating the RAS pathway
Source: J Cell Mol Med. 2021 Aug 26;25(19):9306–18. doi: 10.1111/jcmm.16865 (PMC8500958; doi:10.1111/jcmm.16865)
Supplement: Supplementary file 2 — Table S2 [file JCMM-25-9306-s003.docx]

Table S2 Details of primary and secondary antibodies used for western blotting or immunohistochemical staining.

| **Antibodies** | **CatalogNo.** | **Supplier** |
| --- | --- | --- |
| Rat anti-human HRAS | ab201054 | ABCAM, Inc. UK |
| Rabbit anti-human PLD 1 | ab50695 |  |
| Rabbit anti-human ERK1（pT202/pY204）+ ERK2（pT185/pY187） | Ab4819 |  |
| Rabbit anti-human DUSP 7 | ab95960 |  |
| Rabbit anti-human CASPASE 3 | 196771-ap | Proteintech Inc (USA) |
| Rabbit anti-human CASPASE 7 | Ab32522 | ABCAM, Inc. UK |
| Rabbit anti-human BCL-2 | 12789-1-ap | Proteintech Inc (USA) |
