# Supplementary material for: DUSP7 inhibits cervical cancer progression by inactivating the RAS pathway
Source: J Cell Mol Med. 2021 Aug 26;25(19):9306–18. doi: 10.1111/jcmm.16865 (PMC8500958; doi:10.1111/jcmm.16865)
Supplement: Supplementary file 3 — Table S3 [file JCMM-25-9306-s004.docx]

S_Table 3 Primers for Real-time PCR

| **Gene Name** | | **Primer Sequence** | |
| --- | --- | --- | --- |
|  |  | Forward Primer | Reverse Primer |
|  | **DUSP7** | CTGTCCAGATCCTGCCCTAC | TGGGGATCTGCTTGTAGGTG |
|  | **GAPDH** | CATGAGAAGTATGACAACAGCCT | AGTCCTTCCACGATACCAAAGT |
|  | **CASPASE 3** | AGCCCATTTCTCCATACG | TTATTGCCTCACCACCTTTAG |
|  | **CASPASE 7** | CTGCCTAGTGGGAGTTAGGA | GGATAGGTGAGACCAAGGTA |
|  | **BCL-2** | CTAAGGGTATGAAGGACCTGTA | CTCTGGAATCTAAAGGTCGT |
|  | **GAPDH** | TGTTGCCATCAATGACCCCTT | CTCCACGACGTACTCAGCG |
